# HCCA-SAFE: A Hybrid Cascaded Control Architecture for FPGA-Based Fault Injection in Safety-Critical Automotive SoCs

**DOI:** 10.3390/mi17020185

**Published:** 2026-01-29

**Authors:** Jiajun He, Yuanhao Zhang, Weijie Lu, Yi Liu, Changqing Xu, Xinfang Liao, Yintang Yang

**Affiliations:** 1 School of Microelectronics, Xidian University, Xi’an 710071, China; 2 Guangzhou Institute of Technology, Xidian University, Guangzhou 510555, China; 3 Shenzhen Institute of Technology, Xidian University, Shenzhen 518000, China

**Keywords:** fault injection, FPGA, RISC-V, functional safety

## Abstract

Automotive System-on-Chips (SoCs) must meet stringent functional safety standards, such as ISO 26262 and IEC 61508, to ensure reliable operation under hardware faults. FPGA-based fault injection has emerged as a practical and cost-effective technique for functional safety verification. However, instrumentation-based methods face scalability challenges when applied to the high fault densities typical of automotive SoCs. To address these challenges, we propose a hybrid cascaded fault-injection controller architecture (HCCA-SAFE) that simultaneously reduces high-fanout global nets and eliminates long serial propagation paths. The architecture constrains enable-signal cluster width and distributes control across cascaded stages, improving timing results and routability under limited FPGA resources. The proposed architecture is evaluated on multiple open-source RISC-V processor cores. On openE902, HCCA-SAFE reduces net delay from 27.276 ns to 22.535 ns and achieves 32.2% and 63.8% lower net delay compared with the representative centralized and shift-chain approaches, respectively. On openE906, the proposed HCCA-SAFE limits the net delay to 12.959 ns and reduces the maximum control-signal fanout to 1763, respectively, compared with 25.825 ns and 40.442 ns in the conventional method. On openC906, the proposed design lowers the maximum control-signal fanout from 7725 to 570 and reduces the net delay to 7.506 ns. Furthermore, HCCA-SAFE produces results fully consistent with software-based RTL simulation, while delivering substantial performance gains. Speed-up factors of 127×, 206×, and 2123× are achieved on openE902, openE906, and openC906, respectively, with efficiency improvements scaling with processor complexity These results confirm that HCCA-SAFE delivers scalable, timing-robust fault-injection control suitable for large automotive SoCs.

## 1. Introduction

Electronic systems in automobiles are required to comply with stringent functional safety standards, most notably ISO 26262 [1] and IEC 61508 [2]. A central requirement of these standards is that electronic systems must be capable of preventing failures that could arise from inherent faults. Such faults are typically mitigated or detected through hardware redundancies integrated within automotive System-on-Chips (SoCs), which are referred to as safety mechanisms in ISO 26262. With the rapid growth in the size, architectural complexity, and safety-critical functionalities of automotive SoCs, functional safety verification has become an indispensable requirement for both SoC and IP-level designs. To guarantee that safety goals are met even under the most adverse operating conditions, comprehensive and efficient fault injection campaigns, supported by high-speed simulation, are essential. Despite advancements in commercial fault simulators such as Cadence Xcelium Fault Simulator [3], software-based fault injection methods still struggle to meet the stringent efficiency and scalability requirements inherent to large-scale SoC designs and long-term validation campaigns. To address these challenges, researchers have increasingly turned to hardware-assisted solutions. In particular, Field-Programmable Gate Array (FPGA)-based fault injection techniques have been widely explored in the literature.

There are two primary ways to perform FPGA-based fault injection [4]: reconfiguration-based and instrumentation-based methods. The first method emulates logic faults in FPGA prototypes by dynamically altering the contents of configuration memory (CM) during runtime. However, this method suffers from a lack of direct correlation between the Register Transfer Level (RTL) design nodes and the corresponding bit location of configuration frames, making precise fault targeting difficult [5]. In contrast, the instrumentation-based approach involves modifying the design under test (DUT) by inserting additional logic to simulate fault effects explicitly within the design. Nevertheless, this method incurs additional area and timing overhead [6], and it becomes increasingly impractical when the DUT involves thousands to millions of potential fault sites, due to scalability and resource limitations.

In this paper, we propose a novel **H**ybrid **C**ascaded fault injection **C**ontroller **A**rchitecture for **SAFE** critical (HCCA-SAFE) Automotive SoCs verification that redefines the way control is distributed in FPGA-based fault injection. The proposed architecture introduces a distributed cascaded topology that localizes control distribution while preserving global flexibility. This hybrid design uniquely enables simultaneous improvements in timing performance, scalability, and resource efficiency, addressing a long-standing trade-off in existing solutions. To the best of our knowledge, this is the first architecture that explicitly targets large-scale fault injection campaigns under strict FPGA area and performance constraints, thereby offering a new pathway toward practical and high-throughput fault injection platforms. The remainder of this paper is organized as follows. First, we review the background and related work. Next, the proposed fault injection controller architecture is presented in detail, followed by a description of the fault injection flow. Subsequently, the experimental setup and results are discussed. Finally, conclusions are drawn.

## 2. Background and Related Work

### 2.1. FPGA-Based Fault Injection Methods

There are two main ways to perform FPGA-based fault injection: Reconfiguration and Instrumentation.

Reconfiguration-based approach: The use of partial or full reconfiguration in FPGAs via the internal configuration access port (ICAP) [7] allows designers to change the configuration frames of the DUT, and configure it into faulty states, after which emulation can be performed to analyze the resulting system response. This approach offers the advantage of introducing no additional area overhead, as faults are reproduced directly through configuration.

**Figure 1:**
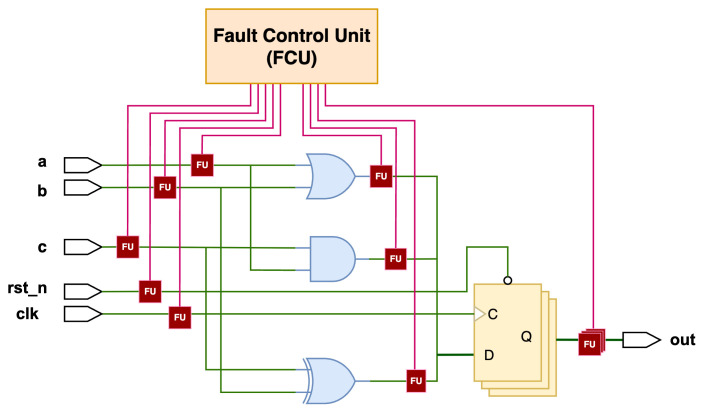
Example of an instrumentation-based fault injection architecture.

Instrumentation-based approach: The DUT circuit is modified by inserting dedicated fault units (FUs), and all the units are controlled by a fault control unit (FCU) to accomplish different fault injection scenarios, as shown in Figure 1. This approach enables rapid fault injection campaigns and can achieve significant speed-ups compared to reconfiguration-based methods.

### 2.2. Fault Models in ISO 26262

Within the standard, two primary categories of fault models are defined to represent random hardware failures: **permanent faults**, such as Stuck-at-1(SA1) and Stuck-at-0(SA1), typically modeled as stuck-at conditions that emulate manufacturing defects or wear-out phenomena; and transient faults, which include Single-Event Upsets (SEUs) and Single-Event Transients (SETs) arising from radiation-induced effects or signal crosstalk. The potential fault injection sites across different design levels, identified in accordance with standard fault models, are summarized in Table 1. Compared with soft-error sensitivity evaluation in space-grade SoCs, functional-safety verification for automotive SoCs must account for a much broader spectrum of fault types. This substantially increases both the number of faults that must be injected and the duration required to complete the verification campaign.

### 2.3. Related Work

As efforts to address the challenges of fault injection efficiency, recent works have primarily focused on using hardware-assisted methods. Most of the work chooses to use the reconfiguration-based method to evaluate the design sensitivity [5,8,9]. However, these methods exhibit several intrinsic limitations when applied to the functional safety evaluation of automotive SoCs. First, the reconfiguration process often becomes a performance bottleneck due to the latency associated with communication between the host computer and the FPGA configuration interface [4]. Second, this method depends on analyzing FPGA bitstream formats, yet the documentation of modern bitstreams is notoriously incomplete and vendor-restricted [5]. Consequently, most reconfiguration-based fault injection studies restrict themselves to random bit-flip emulation at the configuration memory level, without establishing a clear correlation between the injected faulty bits and the behavior of the DUT. This method is scarcely applicable to functional safety verification, as designers must be able to inject faults directly into safety-critical modules rather than relying on random or indirect fault emulation.

**Figure 2:**
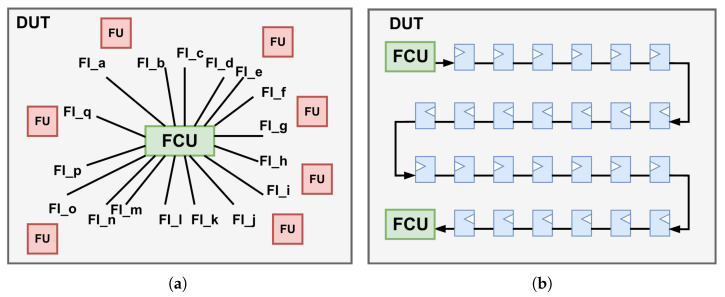
Illustration of two conventional fault injection control architectures. (**a**) Single centralized [12]. (**b**) Fully cascaded [10].

A limited number of studies have explored instrumentation-based methods for design evaluation [10,11,12]. However, these investigations typically consider only a very small subset of faults, with the majority restricted to single-event effect (SEE) sensitivity analysis. Unlike reconfiguration-based methods, the instrumentation-based approach directly alters the DUT. However, its area and timing overhead become particularly critical when the DUT contains a large number of potential fault sites, since the additional instrumentation logic substantially increases design complexity and often makes placement and routing in EDA tools infeasible. Two conventional control architectures are illustrated in Figure 2. The first is a centralized control scheme, in which a single FCU directly manages all fault injection points. While this approach simplifies control logic, it suffers from several critical limitations. Most notably, it introduces extremely high fanout for control signals, resulting in severe routing congestion and significant challenges in achieving timing closure, particularly in large-scale designs. The second is a fully cascaded control architecture that addresses fanout and congestion issues. However, this structure incurs increased control latency due to sequential signal propagation along the cascaded paths.

Table 2 summarizes the major features of recent FPGA-based fault emulators, and our work is highlighted with a gray background. The term “Recon.” denotes reconfiguration-based methods. As shown in the table, the performance of reconfiguration-based approaches typically ranges from tens to thousands of milliseconds, depending on the complexity of the DUT. Due to the huge amount of configuration memory frame bits in complex designs, such as RISC-V cores, reconfiguration-based methods typically rely on statistical fault injection rather than exhaustive injection. The term “Instr.” in the lower half of the table represents the instrumentation-based method. Compared with reconfiguration-based methods, the instrumentation methods achieve injection latencies in the microsecond range, representing an improvement of several orders of magnitude over millisecond-level reconfiguration-based techniques. In the work by Celia López-Ongil et al. [13], two fault control architectures, namely Time-Multiplexed and Shift-Scan, are proposed. However, this study primarily focuses on analyzing soft errors in registers of the DUT. Consequently, the number of fault sites is inherently limited to the registers present in the benchmark circuits, and the scalability of the proposed architectures to larger designs or broader fault models is not addressed. For Felipe Serrano [14], they used a reconfiguration and instrumentation mixed method to control fault injections. While exploiting the flexibility of the ICAP interface, the approach inherits the fundamental drawbacks of reconfiguration-based methods. For EFIC-ME [15] and Zih-Ming Huang [16], both approaches leverage the flexibility of a host PC script to control fault injection. However, this reliance on an external PC inevitably introduces additional communication overhead, which limits the overall injection efficiency and scalability. Compared with recent FPGA-based fault emulators, the proposed HCCA-SAFE introduces a novel hybrid cascaded hardware control architecture that supports diverse fault models and a large number of fault sites, as required by functional safety verification campaigns. Moreover, HCCA-SAFE maintains performance at the same order of magnitude as conventional instrumentation-based methods, even when applied to highly complex designs. To the best of our knowledge, this work presents the first instrumentation-based fault emulator that considers more than one hundred thousand (144,378 = 3 × 48,126) fault sites as required for large-scale functional safety verification.

## 3. A Hybrid Cascaded Fault Injection Controller Architecture

**Figure 3:**
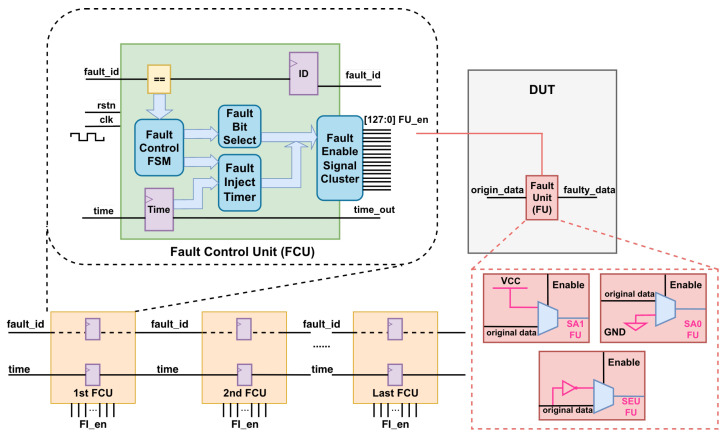
The hybrid cascaded fault injection controller architecture.

To overcome the challenges inherent in the instrumentation-based method, we propose a hybrid cascaded fault injection controller architecture that is optimized for handling a large number of potential fault sites and supporting SA0, SA1, and SEU fault models, thereby improving both scalability and applicability in complex SoC designs. Leveraging a hierarchical and decoupled control design, the proposed architecture reformulates fault injection from a fine-grained instrumentation problem into a scalable control distribution problem. Specifically, the FCU and FU decouple fault model specification from fault execution, allowing fault semantics to be configured independently of injection targets. Under this organization, the FCU centrally manages fault activation logic and distributes control through bounded-width enable clusters, while the FU performs localized fault injection at designated sites. To further enable deterministic fault activation, time registers are introduced as a first-class temporal control mechanism, providing cycle-accurate triggering of fault events. This combination establishes a programmable, time-aware, and scalable fault injection architecture, rather than a simple aggregation of control logic. Figure 3 illustrates the proposed architecture. At the core of the design is the FCU, which integrates a Fault Control Finite State Machine (FSM), a Fault Bit Selection Module, a Fault Injection Timer, and a Fault Enable Signal Cluster.

### 3.1. Microarchitecture

#### 3.1.1. Fault Control FSM

**Figure 4:**
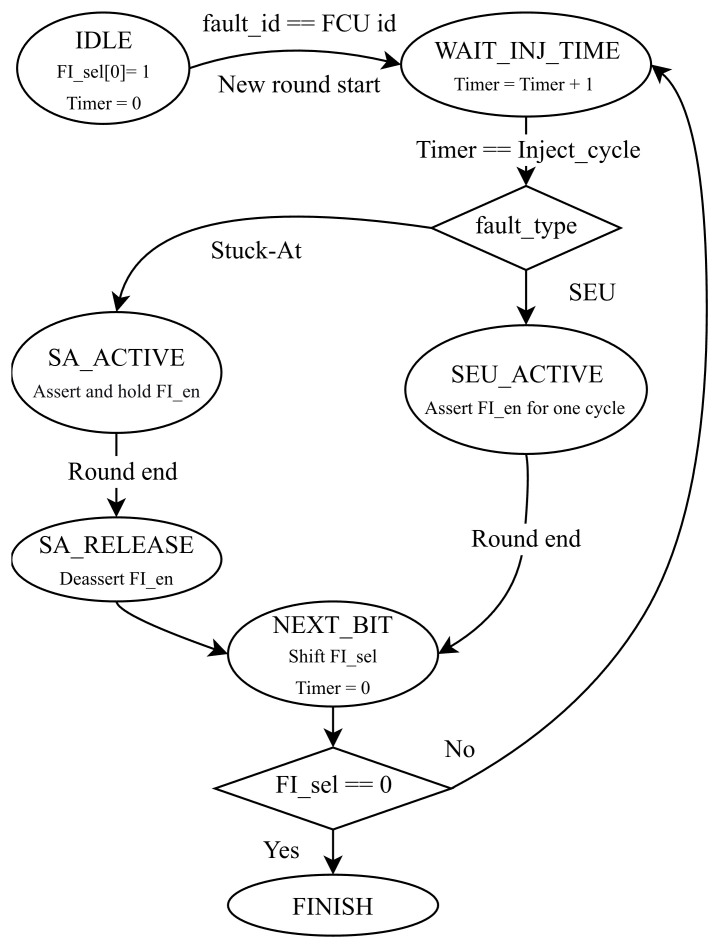
State transition diagram of the fault control finite-state machine.

This component is driven by the system clock and reset signals, and it additionally receives the control signal (nxt_cycle_i) propagated from the preceding FCU. These inputs collectively determine the scheduling and regulation of fault injection events, and the corresponding state-transition diagram of the controller FSM is shown in Figure 4. The FSM will be initialized in the IDLE state after reset and transitions to a wait state upon fault ID matching with a start signal. Once the internal timer reaches the target injection cycle, the FSM executes the injection based on the fault type: generating a single-cycle pulse for SEU or holding the enable signal for SA faults until the round concludes. Afterward, the FSM enters the NEXT_BIT state to reset the timer and left-shift the selection vector; it then loops back to inject the next bit or transitions to FINISH.

#### 3.1.2. Fault Bit Selection Module

Based on the control signals generated by the Fault Control FSM, this module identifies the specific FUs to be activated in a given fault injection campaign. The corresponding enable signals are then propagated to the subsequent stage to ensure correct fault activation and synchronization across the system.

#### 3.1.3. Fault Injection Timer

There is a configuration register to receive the fault injection timing signal (time). Once initialized, the internal timer will autonomously increment with each clock cycle, enabling precise scheduling of FU activation at the target clock cycle prescribed by the fault injection campaign.

#### 3.1.4. Fault Enable Signal Cluster

To minimize the risk of control signals (FU_en) for FUs to become high-fanout nets, which may otherwise degrade timing closure and increase implementation complexity, we constrain the output of the fault enable signal cluster to a maximum width of 128 bits. This value is derived from empirical design experience, balancing scalability and feasibility. In addition, the architecture maintains the parallel enable signal, ensuring flexible and efficient FUs control, particularly in cases where the ordering of fault injection events has not been determined in advance. Although the width of FU_en is limited to 128 bits, fault units are activated in a time-multiplexed manner across multiple injection cycles. As a result, the FU_en can be configured as a one-hot vector when triggering a single fault unit in a fault injection campaign, or as a multi-bit enable vector when multiple fault units are triggered simultaneously.

#### 3.1.5. Fault Unit

To reduce area overhead, the design of the FU prioritizes simplicity and minimal hardware resources. To comprehensively evaluate the proposed architecture, three commonly used types of fault model units are implemented. Each FU modifies the original stream (origin_data) to produce faulty data (faulty_data) when activated.

### 3.2. Latency and Resource Overhead Model

The worst-case control latency model of the proposed hybrid cascaded fault-injection controller can be written as:

(1) $$T_{\max}=H\cdot t_{\mathrm{hop}}$$

where $T_{\max}$ denotes the end-to-end worst-case control latency, $t_{\mathrm{hop}}$ represents the single-hop propagation delay between two adjacent FCU stages ($t_{\mathrm{hop}}$ = 1 cycle in our implementation), and *H* is the hop count that a control signal must traverse in the worst case.

The hop count can be approximated by

(2) $$H=\frac{N_{\mathrm{FU}}}{C}$$

where $N_{\mathrm{FU}}$ is the total number of instrumented FUs and *C* is the number of fault units that a single FCU can control, corresponding to the width of FU_en signal in Figure 3 (C=128 in our implementation).

The resource overhead introduced by the FCUs and FUs can be expressed as follows:

(3) $$R_{\mathrm{total}}=N_{\mathrm{FU}}\cdot r_{\mathrm{FU}}+N_{\mathrm{FCU}}\cdot r_{\mathrm{FCU}}$$

where $R_{\mathrm{total}}$ denotes the overall FPGA resource overhead (e.g., LUTs and FFs). The term $r_{\mathrm{FU}}$ is the per-FU resource cost, which is typically lightweight and often merged with the existing logic of the target design. In contrast, $r_{\mathrm{FCU}}$ represents the fixed per-controller cost of an FCU, including control logic, state registers, and local routing (71 LUTs, 293 FFs, and 6 CARRY8 blocks in our implementation). Overall, the resource overhead scales approximately linearly with the numbers of FUs and FCUs. The FU contribution reflects fine-grained incremental cost, while the FCU contribution introduces coarse-grained but fixed per-stage overhead.

In summary, to scale the architecture for a large number of potential fault sites, multiple FCUs are organized in a cascaded topology, with each unit receiving the cycle control signal from its immediate predecessor. Compared with conventional architectures, the proposed hybrid cascaded structure restricts the width of the fault enable signal cluster to 128 bits, thereby effectively mitigating the risk of high-fanout nets while preserving parallel enable lines to support flexible and simultaneous FUs activation.

## 4. Fault Injection Flow

**Figure 5:**
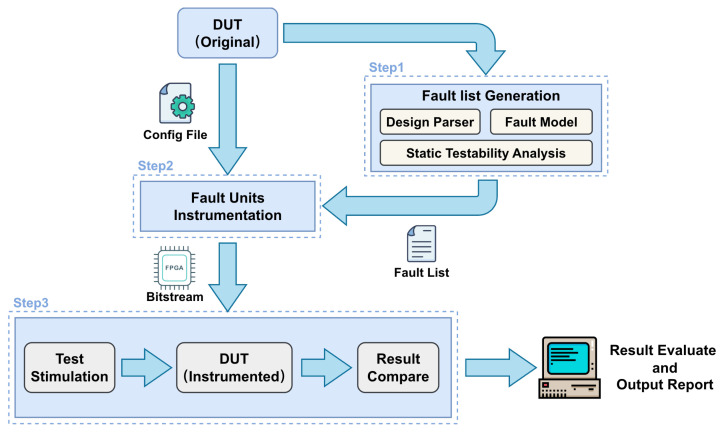
Automated fault injection flow for emulation.

Based on the architecture introduced in Section 3, we establish a fully automated workflow, as illustrated in Figure 5. This workflow consists of three major steps: (i) parsing the DUT design to generate a comprehensive fault list, (ii) performing FUs instrumentation on the DUT, (iii) deploying the instrumented DUT onto the FPGA platform.

### 4.1. Fault List Generation

The workflow begins with the generation of a comprehensive fault list from the original DUT design. This step integrates multiple analysis procedures, including design parsing, fault modeling, and static testability analysis. The design parser extracts fault injection sites information from the DUT, while the fault model defines potential fault scenarios relevant to functional safety verification. Static testability analysis further refines and reorders the fault list by identifying candidate sites with high controllability and observability, and distributing the faults of the same module across appropriate FCU units according to their quantity.

### 4.2. Fault Unit Instrumentation

Once the fault list is established, the next stage focuses on FU instrumentation. As summarized in Algorithm 1, the DUT is automatically modified according to a user-defined constraints file, where specific fault types of FUs (e.g., SA or SEU), and the DUT modules are injected at the designated fault sites. The hybrid cascaded FCUs are instantiated at the top level of the design to coordinate the activation of different FUs. Following instrumentation, the modified DUT undergoes synthesis, placement, and routing to generate the FPGA bitstream.

### 4.3. FPGA Deployment and Experimental Evaluation

The final step involves deploying the instrumented DUT onto an FPGA platform for experimental fault injection. The FPGA-based prototype is subjected to test stimulation, during which input vectors are applied to activate both normal and faulted behaviors. The output of the faulty DUT is compared with the golden DUT directly in hardware on an FPGA platform. The fault injection results are then stored in the on-chip memory and transferred to the host PC after all fault injection campaigns are completed. Finally, the evaluation results are compiled into automatically generated reports, providing quantitative insights into fault coverage, error propagation, and system robustness.
**Algorithm 1** Automated Fault Injection Instrumentation Flow**Require:** Original design *D*, fault list FL, user constraints Cons, FU library $L_{FU}$**Ensure:** Instrumented design $D^{\prime }$, top-level controller $FI_{\mathrm{Ctrl}}$  1: $S_{\mathrm{tgt}}\leftarrow \text{IdentifyCandidates}\left(D,FL,Cons\right)$       ▹Select target signals based on constraints  2: **for all** modules m∈D containing signals in $S_{\mathrm{tgt}}$ **do**  3:    **for all** target signals $s\in (m\cap S_{\mathrm{tgt}})$ **do**  4:     $FU\leftarrow \text{Instantiate}(L_{FU},\text{Type}\left(s\right))$    ▹Instantiate Specific FU based on constraints  5:     InterceptSignal(m,s,FU)     ▹Insert FU between driver and loads  6:    **end for**  7:    $\text{RoutePorts}(m,FU_{\mathrm{ctrl}})$ ▹Aggregate and expose control ports to module boundary  8: **end for**  9: $M\leftarrow \text{BuildHierarchyMap}(D^{\prime })$       ▹Build Hierarchical Mapping Tables10: $FI_{\mathrm{Ctrl}}\leftarrow \text{SynthesizeController}\left(M\right)$  ▹Generate logic to address and control all FUs11: **return**
$D^{\prime }$, $FI_{\mathrm{Ctrl}}$


## 5. Experiment Setup and Results

In this paper, we present two types of fault-injection experiments to evaluate the efficiency of the HCCA-SAFE control architecture. The first campaign targets ISCAS’85 and ISCAS’89 benchmark circuits to evaluate the scalability and resource overhead of the proposed architecture. The second campaign is conducted on two open-source RISC-V processor cores to evaluate the architecture’s applicability and effectiveness in a realistic design scenario, and to compare its performance against conventional approaches such as centralized and shift-chain-based control schemes.

### 5.1. Experimental Setup

In ISCAS and processor campaigns, all designs were synthesised and implemented on an AMD Zynq UltraScale+ FPGA platform (ZCU102; Advanced Micro Devices, Inc., Santa Clara, CA, USA) using Vivado toolchain with the default Synthesis and Implementation strategy (version 2024.1), as shown in Table 3. For the ISCAS fault injection campaign, all effective fault sites in the benchmark circuits were systematically analyzed to provide a full coverage evaluation. In contrast, for the processor core campaign, a full-chip fault injection evaluation would exceed the available FPGA resources on the ZCU102 platform due to the large number of potential fault sites (e.g., 142,095) and the required instrumentation overhead. Therefore, the instruction fetch unit (IFU), a timing-critical and control-intensive module on the processor’s critical path, was selected as a representative target to evaluate the timing overhead introduced by the additional FUs and FCUs. It is important to note that this selection is driven by experimental resource constraints rather than architectural limitations. The proposed fault injection architecture inherently supports full-chip fault coverage, which can be achieved given sufficient FPGA resources through scalable instantiation of the cascaded FCU/FU hierarchy.

#### 5.1.1. ISCAS Benchmark

To assess the scalability and resource overhead of the proposed fault-injection architecture, we performed experiments on seven representative circuits from the ISCAS’85 and ISCAS’89 benchmarks. These designs span a broad spectrum of structural complexity—from small, simple modules to large, complex circuits. The LUT and FF utilization reported by Vivado synthesis tools, together with the potential fault sites of each circuit extracted by our analysis scripts, are summarized in Table 4.

#### 5.1.2. RISC-V Processor

To further evaluate the practical applicability of the proposed fault-injection architecture beyond benchmark-scale circuits, experiments were conducted on three different open-source RISC-V processor cores: openE902 [18], openE906 [19], and openC906 [20]. These processors exhibit distinctly different architectural characteristics, spanning from lightweight embedded cores to high-performance application-class designs. Specifically, openE902 is a 32-bit lightweight embedded core with a shallow two-stage pipeline and a compact microarchitectural organization. openE906 is a 32-bit RISC-V processor configured in this study with a five-stage pipeline, supporting the integer, multiplication/division, atomic, floating-point, and compressed instruction set extensions (IMACF). At the high-performance end, openC906 is a 64-bit application-class processor featuring deeper pipelines and an integrated memory management unit (MMU), enabling the execution of Linux systems. Together, these processor cores form a realistic and structurally diverse evaluation platform that complements the ISCAS benchmark circuits and enables a more comprehensive analysis of the scalability of the proposed fault-injection architecture. In addition, the fault injection efficiency of these processors is evaluated using practical matrix-based workloads, and the results are presented in Section 5.2.4 in detail.

### 5.2. Results

#### 5.2.1. Scalability Analysis on ISCAS Benchmarks

We evaluated the resource overhead using seven representative ISCAS benchmark circuits, whose fault counts range from 374 to 48,126. Table 5 summarizes the incremental LUT and FF utilization, together with the corresponding degradation in maximum operating frequency. As circuit complexity increases, the number of instrumented fault sites grows proportionally, resulting in a consistent upward trend in hardware overhead. The increase in FFs usage is noticeably steeper than that of LUTs. This is because the additional FU combinational logic can be partially merged with existing logic during the synthesis process, thereby limiting LUT expansion. In contrast, the FFs required by the control architecture scale directly with the number of fault sites, leading to a significantly faster growth in FF utilization.

**Figure 6:**
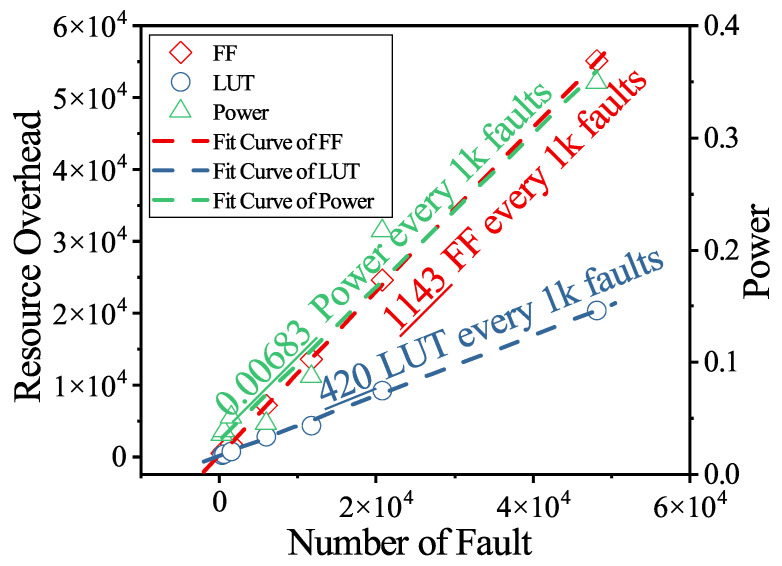
Resource overhead scaling with the number of fault targets.

Figure 6 further illustrates the scalability trend by fitting the LUT and FF overhead as a function of the number of faults, as described in Equation (3). Both resource metrics exhibit a clear linear growth pattern, indicating that the proposed architecture scales proportionally with the number of instrumented fault sites. The fitted models provide a reliable basis for predicting resource utilization for arbitrary fault counts, and they further enable analysis of the maximum number of fault sites that can be supported on a given FPGA platform. The fitted models can be written as:

(4) $$\begin{array}{cc} {\mathrm{LUT}}_{0}+\left(a_{\mathrm{LUT}}N_{f}+b_{\mathrm{LUT}}\right) & \leq {\mathrm{LUT}}_{\mathrm{FPGA}}\\ {\mathrm{FF}}_{0}+\left(a_{\mathrm{FF}}N_{f}+b_{\mathrm{FF}}\right) & \leq {\mathrm{FF}}_{\mathrm{FPGA}} \end{array}$$

where the ${\mathrm{LUT}}_{0}$ and ${\mathrm{FF}}_{0}$ denotes the resource utilization of DUT, and $a_{\mathrm{LUT}}$, $b_{\mathrm{LUT}}$, $a_{\mathrm{FF}}$, $b_{\mathrm{FF}}$ are the fitted parameters of the linear resource models. The ${\mathrm{LUT}}_{\mathrm{FPGA}}$ and ${\mathrm{FF}}_{\mathrm{FPGA}}$ represent the maximum available LUT and FF resources of the target FPGA platform. $N_{f}$ is the number of faults. The maximum of fault sites supported on a given FPGA platform can be expressed as:

(5) $$N_{f}\leq \min\left(\frac{{\mathrm{LUT}}_{\mathrm{FPGA}}-{\mathrm{LUT}}_{0}-b_{\mathrm{LUT}}}{a_{\mathrm{LUT}}},\frac{{\mathrm{FF}}_{\mathrm{FPGA}}-{\mathrm{FF}}_{0}-b_{\mathrm{FF}}}{a_{\mathrm{FF}}}\right)$$

Despite the increase in resource usage, the timing degradation remains within a predictable range. The reduction in maximum frequency varies between −155 MHz and −784 MHz, depending primarily on circuit size and structural depth. Importantly, no abrupt timing collapse or nonlinear bottleneck is observed as circuit size increases, which confirms that the proposed architecture maintains stable and predictable timing behavior when scaling to tens of thousands of faults.

Nevertheless, as an instrumentation-based approach, the area overhead, particularly the FF consumption, scales linearly with the number of fault sites and may become a limiting factor on resource-constrained FPGA platforms. In scenarios involving full-chip fault injection on small or mid-range FPGAs, the proposed approach may therefore be impractical due to area and associated power constraints. The approach is best suited for safety-critical validation scenarios that require high fault coverage, cycle-accurate fault modeling, and fast fault simulation requirements, where the additional area and power overhead represent a deliberate trade-off for accuracy and observability. On platforms with limited resources, practical deployment can be achieved through selective module-level fault injection or reduced fault sites, without architectural modification.

#### 5.2.2. Impact of Cluster Width on Scalability and Implementation

To further investigate the impact of cluster width on timing, fan-out, routing congestion, and resource usage, a simple design space exploration (DSE) experiment is conducted. The results are summarized in Table 6. The maximum achievable clock frequency is jointly influenced by circuit scale, fault count, and the fault-enabled cluster width, exhibiting distinct sensitivity across designs. For the larger s38417 circuit (24,063 fault nodes), the clock frequency peaks at 171.5 MHz with a cluster width of 1024, while comparable frequencies of 141.75 MHz are observed at widths of 128. Similarly, the smaller s15850 circuit (10,399 fault nodes) achieves its highest frequency of 127.54 MHz at a width of 1024, with performance degrading to 55.45 MHz and 121.15 MHz as the width decreases to 128 and 512. Beyond global reset buffers, the fault-enable register width is the second most significant contributor to maximum fan-out, indicating that wider clusters can adversely impact timing due to increased local control fan-out.

Routing congestion correlates with the selected cluster width and circuit scale. For s38417, congestion levels of 3, 6, and 5 are reported at cluster widths of 128, 512, and 1024, respectively, whereas s15850 remains below Vivado’s congestion reporting threshold (Level 3) across 128 and 512 widths. In terms of resource utilization, the cluster width directly determines the number of FCUs, according to Equation (3). A wider cluster width results in fewer required FCUs and consequently lower control-related resource overhead. Based on the observed routing congestion levels, a cluster width of 128 is selected in this work, and all processor-level experiments are conducted using this configuration.

#### 5.2.3. Performance Evaluation on Processor Cores

The proposed architecture is compared with conventional centralized and shift-chain designs across the openE902, openE906, and openC906 processor cores. The corresponding resource usage and performance overhead of each fault-control strategy are reported in Table 7. Our architecture and the best results of different methods are highlighted in bold. For openE902, HCCA-SAFE reduces the Δ*F*_max_ degradation by 94.7% compared with shift-chain, with a resource overhead of 154.7% LUTs and 17.2% FFs, and also improves over the centralized method by 4 MHz. For openE906, the proposed architecture can achieve 23.5% and 27.0% improvements compared with conventional methods. For openC906, the proposed design improves timing by 81.0% compared with shift-chain, and 45.5% compared with centralized. Although the Shift-Chain approach yields the smallest hardware overhead, it also causes the most significant frequency degradation on both processors. Conversely, the proposed architecture requires more resources to implement the distributed control logic, but it consistently achieves the least performance loss, demonstrating superior timing scalability.

The shift-chain architecture is structurally simpler than both the centralized and proposed methods, resulting in lower LUT and FF overhead. However, its serialized flip-flop–based control path introduces a long critical path, as control signals must propagate sequentially across hierarchical boundaries and FPGA tiles. This sequential propagation leads to substantial net delay, which dominates the overall critical path and causes severe performance degradation, as confirmed by the timing results in Table 8. The results of this work are highlighted with an orange background.

**Figure 7:**
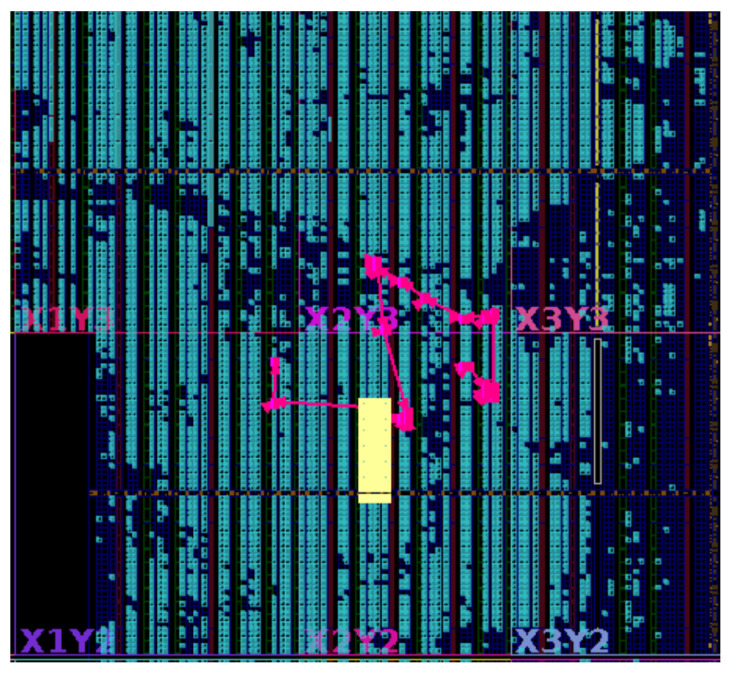
Critical timing path and routing hotspot analysis of the centralized architecture on openC906.

The centralized architecture places all fault control logic in a single module and relies on a wide, high-fanout control signal to activate individual FUs. Although its critical-path timing is better than that of the shift-chain design, the fanout grows rapidly with the number of faults—for instance, reaching 7725 on openC906 compared with 570 in our method—creating severe routing pressure and hotspots. This excessive fanout ultimately degrades timing and limits scalability, even when small designs appear manageable. As shown in Figure 7, the high-fanout signal generates congested routing regions and forces the critical path to traverse multiple overloaded channels, directly contributing to increased net delay and degraded performance.

#### 5.2.4. Efficiency Results on Processor Cores

**Figure 8:**
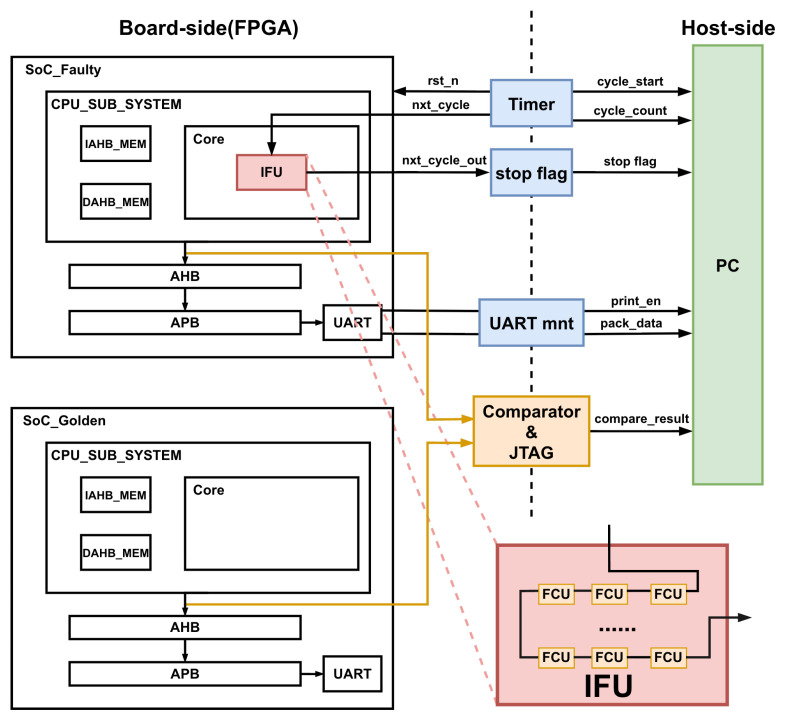
Experimental fault injection platform.

Figure 8 illustrates the fault simulation and verification system constructed for processor-level evaluation. On the FPGA board side, the simple System-on-Chip (SoC) integrates the processor core, on-chip memories, peripherals, fabric bus system, and the proposed fault-injection infrastructure. Faults are injected into the target processor IFU units under the control of the hybrid cascaded FCUs. A cycle-level timer and stop-flag logic are employed to accurately measure the execution latency of each fault injection run.

To measure the fault injection campaign correctness, a fault-free golden SoC is instantiated in parallel with the DUT. Both SoC systems execute the same Matrix workload. During execution, the architectural states and system bus output signals of the faulty SoC and the golden SoC are continuously compared using an on-chip comparator. Any mismatch caused by injected faults is captured and reported through the JTAG interface at the end of all fault campaigns, while detailed execution information is transferred to the host PC.

After the completion of all fault injection runs, the comparison results and timing statistics are collected on the host side for offline analysis. Experimental results show that the outcomes produced by the FPGA-based fault simulation are fully consistent with those obtained from software-based RTL simulation, confirming the functional correctness and reliability of the proposed fault-injection framework. Table 9 summarizes the fault simulation results obtained on the three processor cores.

Table 10 reports the mean time per fault injection run and the corresponding simulation speed-up achieved by the proposed HCCA-SAFE framework, compared with conventional RTL-based fault simulation. For openE902, the average injection time is reduced from 80.7 μs to 0.6367 μs, resulting in a speed-up factor of 127×. Similar trends are observed for openE906, where HCCA-SAFE achieves a 206× acceleration over RTL simulation. The most significant improvement is observed on openC906, for which the average injection time is reduced from 528.6 μs to 0.2490 μs, corresponding to a speed-up factor of 2123×.

## 6. Conclusions

This paper presented HCCA-SAFE, a hybrid cascaded controller architecture that addresses the scalability, timing, and routing bottlenecks of conventional instrumentation-based fault-injection methods. Across two RISC-V processors, the proposed design delivers consistently better overall performance. On openE902, HCCA-SAFE reduces net delay from 27.276 ns to 22.535 ns (17.4%), compared with baseline, and achieves 32.2% and 63.8% lower net delay than the centralized and shift-chain designs. On openE906, the proposed architecture exhibits timing improvements comparable to those observed on openE902. On openC906, it significantly reduces the dominant control-signal fanout to 570, compared with 7725 and 876 in prior methods, and lowers net delay to 7.506 ns. Beyond timing improvements, the proposed framework enables highly efficient and accurate fault simulation on processor-level systems. Experimental results show that the FPGA-based fault simulation outcomes are fully consistent with those obtained from software-based RTL simulation, while achieving substantial acceleration. Specifically, HCCA-SAFE provides speed-up factors of 127×, 206×, and 2123× over RTL-based fault simulation on openE902, openE906, and openC906, respectively, with the efficiency gains becoming more pronounced as processor complexity increases. These results demonstrate that HCCA-SAFE provides superior timing robustness, reduced global fanout, and improved routability, establishing a scalable and efficient foundation for large-scale FPGA-based functional-safety fault injection in automotive SoCs.

## Figures and Tables

**Table 1:** Fault types and injection sites across design levels.

Design Level	Fault Type	Injection Site
RTL level	Permanent	Design module input/output ports, wire, and reg variables
	Transient	reg variables or arrays
Netlist level	Permanent	Standard cell input/output ports, and net variables
	Transient	Sequential cell output ports

**Table 2:** Comparison of recent FPGA-based fault emulator.

FPGA-Based Fault Emulator	Injection Paradigm	FPGA	Performance	Design Under Test	Max Sites	Models	Control Methods
A. Ullah [9]	Recon.	Virtex-5	0.434–7.67 ms/fault	ITC’99, 32/64-bit Adder and Multiplier	94,125	SA0/1	Configuration Memory bit-modify
ACME-2 [17]	Recon.	NA	0.145 s/fault	CORDIC algorithm and NOEL-V processor	101,001/10,000 ^†^	SEU	Configuration Memory bit-flip
BAFFI [5]	Recon.	Virtex Ultrascale+	26–1145 ms/fault	MC8051, AVR, Microblaze and NOEL-V	5000 ^†^	SEU	Configuration Memory bit-flip
Celia López-Ongil [13]	Instr.	Virtex-2000E	0.47–53 μs/fault	ITC’99 Benchmark	582	SEU	Time-Multiplexed and Shift-Scan
Felipe Serrano [14]	Instr.	Virtex-5	1.6 μs/fault	ITC’99 Benchmark	622	SEU	ICAP
FLINT [10]	Instr.	ML605	NA	HC11	∼400	SA0/1 + SEU	Shift-Scan
EFIC-ME [15]	Instr.	Kintex-7	0.39–7.95 μs/fault	32-bit Adder and Shift Multiplier	512	SA0/1 + SEU + MBU	Control Scripts on PC
Zih-Ming Huang [16]	Instr.	NA	3–4 ms/fault	PicoRV32	∼1500	SET + SEU	Control Scripts on PC
HCCA-SAFE	Instr.	Zynq-UltraScale+	249–816 μs/fault	ISCAS’85/89 Benchmark and RISC-V processors ^††^	48,126	SA0/1 + SEU	Hybrid Cascaded Control

^†^ In large-scale designs, the DUT contains a substantial number of configuration bits, making exhaustive fault injection campaigns unfeasible, so statistical injections have been performed. ^††^ The RISC-V cores include openE902, openE906, and openC906.

**Table 3:** Vivado default synthesis and implementation strategy details.

**(a) Synthesis**				
**Strategy**	**directive**	**fsm_extraction**	**keep_equ_regs**	**resource_share**
Default Synthesis	✓Default	✓Auto	×	×
**(b) Implementation**				
**Strategy**	**opt_design**	**place_design**	**phys_opt_design**	**route_design**
Default Implementation	✓Default	✓Default	×	✓Default

**Table 4:** Overview of the benchmark circuits and processors.

Category	Circuits	# Faults ^†^	LUT	FF	Fmax	Power
ISCAS	s349	374	24	15	1144 MHz	0.635 W
	s832	624	59	5	622 MHz	0.635 W
	s1423	1500	106	74	343 MHz	0.629 W
	s5378	5960	254	162	519 MHz	0.670 W
	s9234	11,692	174	136	508 MHz	0.663 W
	s15850	20,798	100	130	781 MHz	0.657 W
	s38417	48,126	1222	1517	319 MHz	0.803 W
Processor	openE902	992	10,782	4472	35 MHz	0.650 W
	openE906	4096	32,878	14,266	125 MHz	0.960 W
	openC906	7717	77,629	30,788	112 MHz	0.951 W

^†^ Fault represents the count of fault sites. The total number of faults can be computed as: Total Faults = Fault Sites × Fault Models per Site.

**Table 5:** ISCAS circuits, resource, and frequency overhead.

Circuit	# FUs	# FCUs	ΔLUT	ΔFF	ΔFmax (MHz)	ΔPower (W)
s349	374	3	+209	+513	−784	+0.035
s832	624	5	+359	+879	−219	+0.039
s1423	1500	12	+723	+1780	−155	+0.051
s5378	5960	47	+2730	+7062	−406	+0.045
s9234	11,692	92	+4347	+13,549	−341	+0.087
s15850	20,798	163	+9147	+24,547	−719	+0.217
s38417	48,126	376	+20,308	+55,134	−237	+0.349

**Table 6:** Different cluster width in s38417 and s15850.

**(a) ISCAS Benachmark s38417 Circuit**			
**Cluster Width**	**128**	**512**	**1024**
$F_{max}$	141.75 MHz	51.27 MHz	171.5 MHz
FF	56,901	51,543	51,693
LUT	21,898	17,787	17,277
Power	1.101 W	1.030 W	0.958 W
Congestion Level	Level 3	Level 6	Level 5
**(b) ISCAS Benchmark s15850 Circuit**			
$F_{max}$	55.45 Hz	121.15 MHz	127.54 MHz
FF	24,759	22,953	23,597
LUT	9261	7634	7516
Power	0.885 W	0.819 W	0.784 W
Congestion Level	<Level 3	<Level 3	Level 5

**Table 7:** Resource and frequency overhead of different fault-control architectures on processor cores.

Processor	Architecture	ΔLUT	ΔFF	Δ*F*_max_ (MHz)	ΔPower (W)
openE902	Centralized [12]	+366	+2100	−5	+0.012
	Shift-Chain [10]	**+358**	**+2053**	−19	**+0.010**
	**This Work**	+912	+2407	**−1**	+0.013
openE906	Centralized [12]	**+5778**	**+7572**	−85	**+0.080**
	Shift-Chain [10]	+14,421	+8834	−89	+0.132
	**This Work**	+6725	9380	**−65**	+0.106
openC906	Centralized [12]	+1908	+15,547	−22	+0.106
	Shift-Chain [10]	**+1894**	**+15,510**	−63	**+0.098**
	**This Work**	+6277	+17,934	**−12**	+0.123

**Table 8:** Timing analysis on all processor cores across different fault-control architectures.

**(a) openE902**				
**Metric**	**Net Delay**	**Logic Delay**	**High Fanout**	**Hotspot**
Baseline	27.276	3.855	cr_cp0_status (555)	N/A
Centralized [12]	33.223	2.956	FICTRL_FI_sel (1275)	N/A
Shift-Chain [10]	62.168	5.092	FSM_onehot_curst (888)	N/A
This Work	22.535	4.857	clic_int_attr (669)	N/A
**(b) openE906**				
Baseline	6.728	1.365	pa_ifu_vec (1795)	N/A
Centralized [12]	25.825	2.775	FCU_cycle (4096)	N/A
Shift-Chain [10]	40.442	3.711	pa_ifu_vec (2123)	N/A
This Work	12.959	2.545	pa_cp0_trap_csr (1763)	N/A
**(c) openC906**				
Baseline	7.070	1.859	ifu_warm (711)	N/A
Centralized [12]	8.340	2.126	FCU_cycle (7725)	X43Y149 (Level 2)
Shift-Chain [10]	19.537	2.233	chain_reg (876)	X51Y253 (Level 1)
This Work	7.506	2.357	FCU_en (570)	N/A

**Table 9:** Software-based and FPGA-based simulation results.

Design	Untestable Faults	Potentially Detected Faults ^†^	Detected Faults	Undetected Faults	Total Faults	Fault Coverage ^††^
openE902 (Software)	13	29	1247	627	1916	66.60%
openE902 (FPGA)	N/A	N/A	1276	640	1916	66.60%
openE906 (Software)	40	21	5664	3953	9678	58.74%
openE906 (FPGA)	N/A	N/A	5685	3993	9678	58.74%
openC906 (Software)	54	0	8624	6810	15,488	56.03%
openC906 (FPGA)	N/A	N/A	8678	6810	15,488	56.03%

^†^ Potentially Detected indicates that the software-based fault simulation produces X-state; however, such a state does not occur in real hardware. Consequently, the corresponding result is reported as N/A in fpga-based emulations. ^††^ Fault Coverage is calculated by $\frac{Detected\;Faults+Potentially\;Detected\;Faults}{Total\;Faults}\times 100\%$.

**Table 10:** Mean time per injection run and estimated speed-up factor.

Design	Mean Time per Injection Run (μs)		Simulation Speed-Up Factor
	RTL	HCCA-SAFE	HCCA-SAFE vs. RTL
openE902	80.7	0.6367	127×
openE906	168.1	0.8160	206×
openC906	528.6	0.2490	2123×

## Data Availability

The data presented in this study are available on request from the corresponding author.
